# Cognitive Food Processing in Binge-Eating Disorder: An Eye-Tracking Study

**DOI:** 10.3390/nu9080903

**Published:** 2017-08-19

**Authors:** Ingmar Sperling, Sabrina Baldofski, Patrick Lüthold, Anja Hilbert

**Affiliations:** 1 Integrated Research and Treatment Center AdiposityDiseases, Leipzig University Medical Center, Medical Psychology and Medical Sociology, Psychosomatic Medicine and Psychotherapy, Leipzig 04103, Germany; Ingmar.sperling@medizin.uni-leipzig.de (I.S.); anja.hilbert@medizin.uni-leipzig.de (A.H.); 2 Department of Psychology, University of Fribourg, Fribourg 1700, Switzerland; patrick.luethold@unifr.ch

**Keywords:** binge-eating disorder, eating disorder, attentional bias, visual, eye-tracking

## Abstract

Studies indicate an attentional bias towards food in binge-eating disorder (BED); however, more evidence on attentional engagement and disengagement and processing of multiple attention-competing stimuli is needed. This study aimed to examine visual attention to food and non-food stimuli in BED. In *n* = 23 participants with full-syndrome and subsyndromal BED and *n* = 23 individually matched healthy controls, eye-tracking was used to assess attention to food and non-food stimuli during a free exploration paradigm and a visual search task. In the free exploration paradigm, groups did not differ in their initial fixation position. While both groups fixated non-food stimuli significantly longer than food stimuli, the BED group allocated significantly more attention towards food than controls. In the visual search task, groups did not differ in detection times. However, a significant detection bias for food was found in full-syndrome BED, but not in controls. An increased initial attention towards food was related to greater BED symptomatology and lower body mass index (BMI) only in full-syndrome BED, while a greater maintained attention to food was associated with lower BMI in controls. The results suggest food-biased visual attentional processing in adults with BED. Further studies should clarify the implications of attentional processes for the etiology and maintenance of BED.

## 1. Introduction

Binge-eating disorder (BED) is the most prevalent eating disorder with high clinical significance [1]. It is associated with increased psychopathology, the impairment of quality of life, and being overweight and obese [2]. However, little is known about the cognitive mechanisms underlying binge-eating behavior. This study sought to investigate an attentional bias towards food stimuli in BED which may have relevance to the maintenance of this disorder [3,4].

BED was recently included in the fifth edition of the Diagnostic and Statistical Manual of Mental Disorders (DSM-5; [2]) as a distinct eating disorder diagnosis. BED is characterized by recurrent episodes of binge eating, involving the consumption of an objectively large amount of food accompanied by a sense of loss of control in the absence of regular inappropriate compensatory behaviors [2]. In various studies, BED was shown to be associated with increased eating disorder psychopathology (e.g., weight and shape concern), general psychopathology (e.g., comorbid depression, anxiety disorder, borderline personality disorder, and substance use disorders), and obesity, providing evidence for its clinical significance [5,6].

The evidence on the underlying cognitive mechanisms of binge-eating behavior remains scarce. Cognitive and behavioral research on eating disorders suggests biases in the visual processing of social-emotional stimuli [7]. Further, a cognitive bias and, more specifically, an attentional bias towards food is considered an important factor in the development of disordered eating behavior [3,8,9]. An attentional bias is defined as a distinctive reaction to disorder-relevant, salient stimuli, for example, food stimuli, compared to neutral stimuli [10,11]. The presence of an attentional bias towards disorder-relevant stimuli was demonstrated, for example, in individuals with substance use disorders, anxiety disorder, and depression [4,12,13]. To investigate an attentional bias towards disorder-relevant stimuli in eating disorders, most previous studies used reaction time-based measures such as the modified Stroop test or the dot-probe task, which measure attention only indirectly and cannot reveal its time course [14,15,16,17]. More recent studies used eye-tracking paradigms which measure attention directly, allowing for the measurement of fixation times and the differentiation between early and late stages of attentional processing [3,8]. Thus far, only a few studies using eye-tracking technology have investigated processing of food stimuli in eating disorders and obesity and have yielded inconsistent results.

One study investigating food processing in BED in a free exploration paradigm and an anti-saccadic task yielded an attentional maintenance bias towards food compared to non-food stimuli in women with BED and obesity, compared to women with obesity and women with normal weight [18]. Another eye-tracking study showed an early orientation bias and an overall increased attentional maintenance towards high-and-low-calorie food in real-world scenes in a group of individuals with binge eating, but not BED, and normal weight of both genders compared to the control group [19]. Further, a study in adolescents with BED showed no early orientation bias, but an overall increased attentional maintenance to food stimuli in a free exploration paradigm, as well as faster detection of food compared to non-food stimuli in a visual search task in participants with BED compared to controls [20]. In two studies comparing participants with obesity and normal weight, an early attentional bias towards food stimuli compared to non-food stimuli was reported for participants with obesity during a free exploration paradigm [21,22], followed by a prolonged gaze time towards food stimuli in participants with obesity [21]. However, in either study, a visual dot-probe task combined with eye-tracking did not yield any differences between participants with obesity and normal weight [21,22]. In another study, participants with normal weight showed an attentional bias for food compared to non-food stimuli in a visual search task, but not in a free exploration paradigm [23].

Overall, in a limited number of studies, BED and obesity seem to be characterized by an attentional approach–approach pattern; i.e., rapid orienting and maintained attention towards food stimuli [3]. However, the generalization of previous eye-tracking results in BED is limited insofar as most studies examined only women with BED and obesity [18] and adolescents with BED [20], respectively. BED, although associated with obesity, also occurs in individuals with normal weight, as well as in men [5]. Therefore, an investigation of attentional processes in an adult sample of both genders with BED and with a wider weight-range seems necessary. Further, more evidence on initial attentional engagement and subsequent maintenance or disengagement processes as well as processing of multiple attention-competing stimuli in BED is needed.

The present study aimed to investigate food processing in BED using a free exploration paradigm and a visual search task in a sample of both genders, with body mass indices (BMI, kg/m^2^) ranging from normal weight to obesity. In accordance with a previous eye-tracking study in adolescents with BED from our group [20], we hypothesized an attentional bias towards food stimuli compared to non-food stimuli in adults with BED in comparison to a matched control group. Specifically, we assumed (1) a facilitated initial fixation and longer overall gaze duration on food stimuli in a free exploration paradigm and (2) faster detection of food stimuli among non-food stimuli than vice versa in a visual search task in participants with BED compared to the control group.

## 2. Materials and Methods

### 2.1. Ethics Statement

Written informed consent was obtained prior to study participation. The study was approved by the Ethics Committee of the Medical Faculty of the University of Leipzig (ethical approval code: 180-12-ff-21052012; August 16, 2012).

### 2.2. Participants

Participants in the BED group (*n* = 23) were diagnosed with full-syndrome BED (*n* = 17; 73.9%) according to the DSM-5 [2] or subsyndromal BED (*n* = 6; 26.1%) [24]. The control group (CG, *n* = 23) was individually matched according to sex, age, and BMI (kg/m^2^) and comprised participants without eating disorder symptoms.

Eating disorder diagnosis was based on the DSM-5 criteria for BED [2]. In addition, as this study was planned before the DSM-5 criteria for BED were published, subsyndromal BED included the modified criteria of two instead of three or more behavioral indicators or lack of substantial distress [24]. Exclusion criteria in both groups comprised non-corrected impaired vision, current intake of psychotropic medication or medication affecting weight or eating behaviour, current substance abuse or addiction, psychotic disorder, bipolar disorder, neurological disease, pregnancy or lactation, and at least one episode of inappropriate compensatory behavior within the last three months. Further, in the CG, objective binge-eating episodes within the last three months were an exclusion criterion.

All participants were at least 18 years of age and were required to have sufficient German language skills. They were recruited from the community and received financial compensation for participation. As an initial step, a telephone interview was conducted to check eligibility. To prevent potential effects of hunger or recent food consumption on attentional processing of food stimuli [21,25], eligible participants were instructed to be satiated when arriving at the laboratory, but to refrain from food consumption and caloric drinks one hour prior to the appointment. Using the diagnostic version of the semistructured eating disorder examination (EDE) interview [26,27] diagnostic criteria were assessed at the study appointment. All participants were naive to the purpose of the experiment.

### 2.3. Eye-tracking and Experimental Design

#### 2.3.1. Free Exploration Paradigm (Task 1)

All experimental procedures are described in detail elsewhere and have been previously used in adolescents with BED (see [20]). In Task 1, 30 pairs of matched food and non-food stimuli were randomly presented in a total of 30 trials. The participants were instructed to explore the stimuli as if they were watching television while their eye movements were continuously recorded.

#### 2.3.2. Visual Search Task (Task 2)

Stimuli were presented in a circular search array of three or six food and/or non-food stimuli. The participants were instructed to indicate as quickly and accurately as possible whether the search array contained only stimuli of the same category or whether one stimulus (target) was different from the others (distractors) by pressing one of two pre-specified keys. Overall, the task consisted of four trial types: (1) food target trials: a food target among corresponding matched non-food distractors; (2) non-food target trials: a non-food target among corresponding matched food distractors; (3) food only: only food stimuli; and (4) non-food only: only non-food stimuli, where the same food or non-food stimulus, respectively, was presented on all positions of the array. Analyses included only food target and non-food target trials for hypothesis testing.

#### 2.3.3. Stimuli

The stimulus material for both tasks consisted of 30 food stimuli depicting low to high-caloric foods and 30 non-food stimuli depicting everyday objects which were not related to eating or food. Each food stimulus was matched with a similar non-food stimulus for color, shape, size, and visual complexity. The same stimulus material was used for both tasks.

#### 2.3.4. Apparatus

A desktop-mounted, video-based infrared eye-tracking system was used (Eyelink I, SR Research, Ottawa, ON, Canada) with a spatial resolution of 0.1° and a temporal resolution of 500 Hz. Participants could move their head freely; however, they were instructed to keep it as still as possible at a distance of 60 cm of the display. 

#### 2.3.5. Data Preparation

Experimental trials were valid if (a) the gaze was directed at the fixation cross at trial onset; (b) saccades occurred at least 150 ms after image onset and before image offset; (c) fixations were directed at images (not remaining in the middle); and (d) fixations remained stable within 1° for at least 100 ms.

For Task 1, participants with a total gaze duration 3 standard deviations (SD) below the group’s mean and participants with invalid trials 3 SD above the group’s mean were excluded from the analysis due to lowered data quality [20]. Following these criteria, 952 (69.0%) valid trials were recorded across all participants in the free exploration paradigm and no participant was excluded from the analysis.

For Task 2, trials with incorrect responses and with reaction times of 3 SD below or above the group’s mean across all trial types were excluded [20]. In the visual search task, 7794 (98.4%) valid trials were recorded across all participants. One participant in the CG was excluded as data were not recorded due to technical problems. Due to the individual matching design, the counterpart of this participant was excluded from the analyses as well, resulting in a sample size of *n* = 22 in the BED group and *n* = 22 in the CG, respectively.

### 2.4. Measures and Variables

#### 2.4.1. Attentional Bias Scores

##### Free exploration paradigm (Task 1)

For hypothesis testing, two bias scores were defined: gaze direction bias and gaze duration bias [20,22]. The gaze direction bias score (in %) indicates an initial attentional orientation. It was calculated by computing the number of first fixations directed to the food stimulus as a proportion of all trials in which first fixations were made to either stimulus. An initial orienting bias to food or non-food stimuli is reflected by gaze direction bias scores of > and <50%, respectively, while a bias score of 50% indicates no bias. The gaze duration bias score (in ms) reflects maintenance of attention. It was calculated by subtracting the mean gazing time at non-food stimuli from the mean gazing time at food stimuli [20,22], i.e., positive bias scores reflect longer maintained attention on food than on non-food stimuli, while negative scores indicate longer attentional maintenance on non-food stimuli.

##### Visual search task (Task 2)

By subtracting the mean reaction time on food target trials from the mean reaction time on non-food target trials [20,28], a food detection bias score (in ms) was computed. Positive bias scores indicate faster detection of food stimuli and/or delayed disengagement from food distractor stimuli.

#### 2.4.2. Variables for Clinical Associations

##### Eating Disorder Examination-Questionnaire (EDE-Q)

To assess eating disorder psychopathology, a subset of 22 items assigned to four subscales (restraint, eating concern, weight concern, and shape concern) of the German version of the EDE-Q [29,30] was administered. A global mean score was computed, with higher scores indicating higher levels of eating disorder psychopathology. The EDE-Q showed good reliability, stability, convergent, discriminant, and factorial validity [30].

##### Patient Health Questionnaire—Depression (PHQ-D)

Depressive symptoms were assessed using the German version of the 9-item PHQ short version for depression [31,32]. A higher sum score indicates more depressive symptoms. The PHQ-D showed good reliability and validity [33].

##### Hunger rating

Using a Likert scale ranging from 0 = *not at all hungry* to 6 = *very hungry*, participants rated their hunger prior to the experimental tasks.

##### Valence rating of food stimuli

All food stimuli were presented randomly on the computer screen after the experimental tasks and their pleasantness was rated on a visual analogue scale, resulting in a score from 0 = *not at all pleasant* to 400 = *very pleasant*. An exploratory analysis investigated whether attentional bias scores were driven by stimulus valence; i.e., by participants’ individual ratings of food stimuli. Two categories were created based on a median-split of each participant’s ratings, personally attractive and unattractive food stimuli. Based on these categories, all bias scores were additionally calculated separately for trials including attractive or unattractive food stimuli, respectively. For one participant in the BED group and one participant in the CG, data on valence ratings were not recorded due to technical problems. Accordingly, the counterpart of each participant was excluded as well due to the individual matching in all analyses including valence ratings.

##### Sociodemographic variables

Demographic information obtained included sex, age, and educational level. BMI (kg/m^2^) was calculated from measured weight and height.

### 2.5. Data Analytic Plan

Sample characteristics and group differences in psychopathology, hunger, and valence ratings of food were examined using repeated measures general linear model analyses for continuous (age, BMI, and psychological measures) and *χ^2^* tests for categorical variables (sex and educational level). Group differences in bias scores were analyzed using repeated measures general linear model analyses with group (BED, CG) as within-subject factor to account for the individual matching. As BED group and CG differed significantly in depressive symptoms, hunger levels, and valence ratings of food stimuli, these variables were included as covariates in the analyses in an additional step. One sample *t* tests were used for testing bias scores against 50% (gaze direction bias score) and zero (gaze duration and food detection bias scores), respectively, in each group separately. All dependent variables were normally distributed, and sphericity assumptions were met. Pearson’s *r* correlations between attentional bias scores and study measures were computed in the BED group and CG separately. All analyses were performed (1) in the total study sample and (2) in a subgroup of participants with full-syndrome BED (*n* = 17) compared to their respective matched CG counterparts. The results of the second step were only reported if they differed from the results of the first step.

Based on the reported results of one primary outcome measure (i.e., food detection bias), a post-hoc power analysis was performed, indicating that the group sizes of *n* = 23 in each group obtained 82.1% power to detect differences in food detection bias scores when employing a two-tailed statistical significance criterion of 5% [34]. For effect size estimation, φ was reported for the *χ^2^* tests (small: φ = 0.1, medium: φ = 0.3, large: φ = 0.5), partial η^2^ was reported for the general linear model analyses (small: η^2^ = 0.01, medium: η^2^ = 0.06, large: η^2^ = 0.14), and Pearson’s *r* was interpreted as small, *r* = 0.10, medium, *r* = 0.30, and large, *r* = 0.50 [35]. A two-tailed α = 0.05 was applied to statistical testing. Statistical analyses were performed using IBM SPSS Statistics version 20.0 (IBM Corp., Armonk, NY, USA).

## 3. Results

### 3.1. Sample Characteristics

BED group and CG did not differ regarding sex, age, BMI, and educational level (all *p* > 0.05; see Table 1). Participants with BED reported significantly more OBEs and higher levels of eating disorder psychopathology (*p* < 0.001) and hunger (*p* < 0.01) than the CG. Further, the BED group reported significantly more depressive symptoms (*p* < 0.001) and lower valence ratings of food stimuli (*p* < 0.05) than the CG.

### 3.2. Task 1: Free Exploration Paradigm

#### 3.2.1. Gaze Direction Bias

The BED group and CG did not differ in their initial gaze direction bias in trials presenting food stimuli in general and trials including attractive and unattractive food stimuli only (all *p* > 0.05; small effect sizes; see Table 2). One-sample *t* tests showed that the gaze direction bias scores in each group did not significantly differ from a test score of 50% (all *p* > 0.05; see Table 2).

#### 3.2.2. Gaze Duration Bias

Repeated measures analyses of variance (ANOVAs) revealed significant group differences in gaze duration bias scores. While both groups tended to overall fixate on non-food stimuli longer than food stimuli, participants with BED allocated more attention towards food stimuli in general, as well as attractive and unattractive food stimuli, respectively, than the CG (large effect sizes). Further, in the CG, one-sample *t* tests showed that the gaze duration bias scores differed significantly from zero in trials presenting food stimuli in general and trials presenting attractive and unattractive food stimuli. In the BED group, a significant gaze duration bias was only present in trials including unattractive food stimuli.

For Task 1, all results did not differ when analyzing a subgroup of participants with full-syndrome BED (gaze direction bias: small effect sizes; gaze duration bias: large effect sizes), with the exception of the gaze duration bias test against zero in the BED subgroup in trials including unattractive food stimuli yielding only a marginally significant result (*p* = 0.058).

### 3.3. Task 2: Visual Search Task

#### Food Detection Bias

BED group and CG did not differ in food detection bias scores in trials presenting food target stimuli in general and trials including attractive and unattractive food target stimuli (all *p* > 0.05). However, in the subgroup of participants with full-syndrome BED, analyses indicated a marginally significant tendency for the BED group to detect food target stimuli faster than the CG in trials presenting food targets in general, *F*(1, 15) = 4.27, *p* = 0.056, η^2^ = 0.22, large effect size. This effect was significantly pronounced in trials with unattractive, *F*(1, 14) = 5.46, *p* = 0.035, η^2^ = 0.28 (large effect size), but not attractive food target stimuli, *F*(1, 14) = 0.18, *p* = 0.677, η^2^ = 0.01 (small effect size). While one-sample *t* tests in the total sample showed that the food detection bias scores in each group did not significantly differ from a test score of zero (all *p* > 0.05), additional subgroup analyses revealed significant effects: In participants with full-syndrome BED, food detection bias scores significantly differed from zero in trials presenting food target stimuli in general, *t*(15) = 3.09, *p* = 0.007, and trials including unattractive food target stimuli, *t*(14) = 2.68, *p* = 0.018, but not in trials with attractive food stimuli, *t*(14) = 0.02, *p* = 0.981. In the respective CG, bias scores were not significantly different from zero; all food stimuli: *t*(15) = −0.05, *p* = 0.959; attractive stimuli: *t*(15) = 0.68, *p* = 0.507; unattractive stimuli: *t*(15) = −1.41, *p* = 0.179.

For both tasks, all reported results did not differ when controlling for depressive symptoms, hunger levels, and valence ratings of food stimuli as covariates.

### 3.4. Clinical Associations

The gaze direction and food detection bias scores were not significantly associated with any of the sociodemographic or clinical variables in the BED group or CG of the total sample (all *p* > 0.05). However, in the subgroup with full-syndrome BED, but not in the respective CG, the gaze direction bias showed a marginally significant medium-size negative association with BMI, *r* = −0.44, *p* = 0.081, as well as a medium-size, non-significant positive association with objective binge-eating episodes, *r* = 0.39, *p* = 0.125. In the CG, the gaze duration bias was negatively associated with BMI, *r* = −0.51, *p* = 0.013 (large effect size), and the EDE-Q global score, *r* = −0.43, *p* = 0.041 (medium effect size), but no significant associations emerged in the BED group (all *p* > 0.05).

## 4. Discussion

The present study investigated attention allocation towards food stimuli in participants with BED compared to a healthy control group using eye-tracking. Contrary to our hypothesis, participants with BED showed neither preferential initial orientation to food vs. non-food stimuli nor a longer overall gaze duration towards food vs. non-food stimuli compared to controls in the free exploration paradigm. Results of the visual search task confirmed our hypothesis in a subgroup of participants with full-syndrome BED, which detected food target stimuli faster than the control group.

In the free exploration task, neither the BED nor the control group showed a gaze direction bias to food stimuli. The absence of group differences in participants’ first fixations is in accordance with other eye-tracking studies in adult [18] and adolescent BED [20], respectively. However, preferential initial fixations on food as compared to controls were found in adults with binge-eating episodes in an eye-tracking study [19] and in adults with BED in a study using reaction time-based measures [36]. The differing results might be explained by heterogeneous sample characteristics (e.g., clinical vs. non-clinical samples) and the use of different experimental procedures (eye-tracking vs. reaction time-based measures) and stimulus sets (e.g., images of single foods or objects vs. complex real-world scenes [19]) across the studies. Regarding attentional maintenance, both groups tended to overall fixate on non-food stimuli longer than food stimuli, while participants with BED allocated significantly more attention towards food stimuli than the control group. These results are in accordance with eye-tracking studies in participants with BED and obesity compared to participants with obesity and controls with normal weight during a free exploration paradigm [18] and in participants with binge-eating episodes demonstrating biased attention towards food compared to healthy controls [19]. A study in adolescents with BED using the same experimental set up as in our study yielded similar results, showing no differences in initial gaze direction between groups, but longer gaze duration on food stimuli in the BED than in the control group in the free exploration paradigm [20].

The lack of a gaze direction bias towards food stimuli and the longer overall gaze duration on non-food stimuli in BED might be explained by the recently introduced concept of motivational ambivalence, i.e., an approach-avoidance conflict regarding food stimuli in BED [37]. As assessed by self-report, individuals with BED and who were overweight rated food stimuli significantly more positively than control groups, whereas indirect evaluation of food stimuli via facial electromyography, i.e., recording of involuntary muscle activation during stimulus presentation, turned out to be negative in both the BED and control groups [37]. This approach-avoidance conflict, indicated by positive self-report but negative facial electromyography, was most noticeable in participants with BED [37]. Another recent study also reported a visual approach-avoidance pattern towards food stimuli in a clinical sample of participants with binge-eating behaviors and severe obesity during a visual probe task [17]. In the present study, food stimuli were rated less attractive by the BED than by the control group. In addition, attention was directed longer towards non-food stimuli in both groups during the eye-tracking paradigm, which might be interpreted as avoidance of food stimuli [37]. However, the BED group looked longer at food stimuli than controls, which might indicate an approach component [37]. Further, correlational analyses in our study showed that in the subgroup with full-syndrome BED, lower BMI and more objective binge-eating episodes were associated with an increased initial attention towards food stimuli. These results suggest that higher BED symptomatology, i.e., more objective binge-eating episodes, is related to an approach pattern towards food in early stages of attentional processing.

Several possible explanations for an avoidance behavior regarding food stimuli could be taken into account. As participants with BED and who were overweight were confronted with palatable food pictures during the study session, negative food-related associations such as feelings of shame [38], guilt, and weight gain could have been triggered [39]. The finding that participants with BED looked longer at food than controls might be explained by simultaneously-activated associations of positive reinforcement related to food consumption [40]. Further, personal traits such as dietary restraint, known to be associated with BED [41,42], could have induced the avoidance of food stimuli in BED. Moreover, when being under observation during the study session, participants may have responded in a way which they might have considered socially appropriate (looking away from food stimuli which are negatively associated with unhealthy nutritional properties and overweight [39], towards neutral stimuli). Furthermore, attention allocation in both groups might have been influenced by the attractiveness of the non-food stimuli, which—as a limitation of this study—has not been rated by the participants. Specifically, certain non-food stimuli may have appeared more attractive to participants than the respective matched food stimuli, which might have resulted in the observed gaze duration bias towards non-food. Regarding the control group, previous research reported biased attention towards food stimuli in healthy persons [21,23]. A possible explanation for the different results in our study might be the low hunger levels in the control group, as studies comparing participants under conditions of hunger and satiety showed that attentional biases towards food are more likely to occur in hungry than in satiated individuals [21,25,43].

The visual search task was conducted for the first time in adults with BED, permitting insights in attentional processing in BED when presented with multiple attention-competing food and non-food stimuli. The results confirmed our hypotheses in part, as only in a subgroup with full-syndrome BED, forming the majority of the experimental group (*n* = 17; 73.9%), a marginally significant tendency for the BED group to detect food target stimuli faster than the control group was found. This effect was significantly pronounced in trials with unattractive but not attractive food target stimuli. Our results are in line with previous studies using reaction time-based paradigms to investigate food-biased attention in adults with BED [16,36,44,45,46] and in obesity [47]. Again, the findings of a study in adolescents with BED reporting faster detection of food vs. non-food targets compared to matched controls in a reaction time-based paradigm were confirmed [20].

A visual search paradigm has already been used in healthy students, yielding a detection advantage for food among neutral stimuli only when the stimuli were visually dissimilar; i.e., not matched for specific visual features [23]. However, the detection advantage disappeared when food and neutral stimuli where matched for shape and color [23], indicating that the ability of the visual system to rapidly distinguish food from non-food stimuli seems to be adversely affected when detection-enhancing visual features, such as color and shape, define the appearance of both food and non-food stimuli [23]. In the present study, contrary to healthy students [23], a detection bias for food vs. non-food stimuli was observed in individuals with full-syndrome BED although all stimulus pairs were closely matched for color, shape, size, and visual complexity. It could therefore be assumed that food stimuli are more attractive to individuals with BED than to healthy individuals with normal weight and that the visual system in BED is more effectively able to recognize food among non-food distractors.

Strengths of this study include the diverse sample of participants with BED comprising both genders with a wide range of age and weight, being individually matched with healthy controls, and the use of a clinical interview [26,27] for BED diagnosis according to DSM-5 [2]. Further, participants were unaffected by psychotropic drugs, ruling out the possibility of influences on eating behavior or altered cognitive processes [48]. As mentioned above, a limitation to this study is the lack of a valence rating of non-food stimuli, which would have allowed to examine possible effects of the pleasantness of non-food stimuli on attentional processing. As the study suggests a potential influence of weight status on attentional patterns in BED, a further limitation is the lack of control groups with normal weight with and without BED. The use of control groups with normal weight might contribute to differentiate effects of obesity and BED, respectively, on attentional food processing.

## 5. Conclusions

Taken together, the findings of this study point to differences between individuals with BED and weight-matched controls without eating disorders in attention allocation towards food and non-food stimuli. First, gaze duration on food vs. non-food stimuli was prolonged in BED compared to controls in the free exploration task, while both groups overall allocated more attention towards non-food than food stimuli. Second, individuals with full-syndrome BED were faster than controls in detecting food targets among non-food distractors in the visual search task, reproducing the findings of a recent study using identical experimental procedures in adolescents with BED [20]. Overall, the results provide evidence for biased information processing, specifically selective visual attention to food, in individuals with BED. Research suggests that changes in the selective attentional patterns in psychological disorders have an impact on symptom severity, indicating that attentional biases may causally contribute to psychological dysfunction such as disordered eating behavior [10]. Recent studies point to the potential therapeutic value of cognitive bias modification (CBM) techniques which were developed to directly alter attentional and interpretive biases using computerized training [10]. CBM techniques targeting attentional biases have proven effective in the treatment of anxiety disorders, depression, pain and addictive disorders and may also be used to influence attentional patterns in BED and thus, improve BED symptomatology [10]. In addition, as found in addictive disorders [49], future studies might investigate whether attentional biases to food stimuli in BED can serve as indicators for eating disorder severity and whether an attentional bias reduction can be used as a treatment efficacy measure in BED [50]. Our study highlights the importance of further clarifying the interactions between attentional processing of food stimuli and BED symptomatology as well as the impact of attentional processes on the etiology and maintenance of BED, for example by examining a larger sample with full-syndrome BED in longitudinal studies. Finally, ecological validity in future research might be increased by investigating attentional processing of foods preferentially consumed during binge-eating episodes.

## Figures and Tables

**Table 1:** Sample characteristics and group differences in psychopathology, hunger, and valence ratings of food stimuli.

	BED (*n* = 23)	CG (*n* = 23)	Test *χ^2^* (1)	Effect Size
Sex (*n* female, %)	15 (65.2)	15 (65.2)	0.00	φ = 0.00
Education (≥12 years *n*, %)	19 (82.6)	14 (60.9)	2.68	φ = 0.24
	M (±SD)	M (±SD)	*F*(1, 22)	η^2^
Age	35.30 (11.39)	35.96 (12.20)	0.84	0.04
Body mass index (kg/m^2^)	32.40 (9.24)	32.79 (9.01)	0.59	0.03
EDE Objective binge-eating episodes (*N*)	2.59 (1.92)	0.00 (0.00)	41.98 ***	0.66
EDE-Q Global score (0–6)	2.65 (1.20)	1.10 (1.03)	29.72 ***	0.58
PHQ-D Depression (0–27)	9.59 (4.15)	3.22 (3.62)	25.41 ***	0.54
Hunger Rating (0–6)	0.50 (0.80)	0.04 (0.21)	8.33 **	0.28
Valence Rating Food (0–400)	234.30 (59.23)	364.47 (246.13)	5.87 *	0.23

BED = binge-eating disorder (including full-syndrome and subsyndromal diagnosis); CG = control group without eating disorder symptoms (i.e., no objective binge-eating episodes; no inappropriate compensatory behavior); M = mean; SD = standard deviation; EDE = Eating Disorder Examination; EDE-Q = Eating Disorder Examination-Questionnaire; PHQ-D = Patient Health Questionnaire. For all measures, higher scores indicate higher levels of psychopathology, hunger, and valence, respectively. Objective binge-eating episodes include mean episodes per week over the last three months. * *p* < 0.05; ** *p* < 0.01; *** *p* < 0.001.

**Table 2:** Attentional bias scores as a function of group status.

	BED	CG	Test for Group Differences			Test against 50%/Zero (BED)		Test against 50%/Zero (CG)	
	M (SD)	M (SD)	*F*(df)	*p*	η^2^	*t*(df)	*p*	*t*(df)	*p*
**Free exploration paradigm**									
*Gaze direction bias (%)*									
All food stimuli	53.45 (12.02)	52.42 (10.51)	*F*(1, 22) = 0.11	0.749	0.01	*t*(22) = 1.37	0.183	*t*(22) = 1.10	0.283
Attractive food stimuli	52.34 (15.25)	50.92 (12.93)	*F*(1, 20) = 0.00	0.953	0.00	*t*(21) = 0.72	0.480	*t*(21) = 0.33	0.742
Unattractive food stimuli	49.81 (11.49)	52.72 (9.84)	*F*(1, 20) = 0.35	0.562	0.02	*t*(21) = −0.08	0.939	*t*(21) = 1.30	0.209
*Gaze duration bias (ms)*									
All food stimuli	−81.39 (306.69)	−349.09 (236.06)	*F*(1, 22) = 13.42	0.001	0.38	*t*(22) = −1.27	0.216	*t*(22) = −7.09	<0.001
Attractive food stimuli	−65.14 (345.36)	−269.86 (250.30)	*F*(1, 20) = 5.49	0.030	0.22	*t*(21) = −0.89	0.386	*t*(21) = −5.06	<0.001
Unattractive food stimuli	−138.73 (272.16)	−408.64 (317.01)	*F*(1, 20) = 10.69	0.004	0.35	*t*(21) = −2.39	0.026	*t*(21) = −6.05	<0.001
**Visual search task ^a^**									
*Food detection bias (ms)*									
All food stimuli	7.82 (37.36)	−4.59 (37.59)	*F*(1, 21) = 1.51	0.232	0.07	*t*(21) = 0.98	0.338	*t*(21) = −0.57	0.573
Attractive food stimuli	−8.42 (54.40)	−5.89 (77.14)	*F*(1, 20) = 0.00	0.997	0.00	*t*(20) = −0.71	0.486	*t*(20) = −0.36	0.724
Unattractive food stimuli	18.67 (56.26)	−14.83 (48.70)	*F*(1, 20) = 2.95	0.101	0.13	*t*(20) = 1.52	0.144	*t*(20) = −1.43	0.168

BED = binge-eating disorder (including full-syndrome and subsyndromal diagnosis); CG = control group without eating disorder symptoms (i.e., no objective binge-eating episodes, no inappropriate compensatory behavior); M = mean; SD = standard deviation. Group differences in bias scores were analyzed. In addition, attentional bias scores for all food stimuli were tested against 50% (gaze direction bias score) and zero (gaze duration and food detection bias scores), respectively, for each group separately. ^a^ Reduced sample size due to missing data.
